# Establishment of RNA Interference Genetic Transformation System and Functional Analysis of FlbA Gene in *Leptographium qinlingensis*

**DOI:** 10.3390/ijms241613009

**Published:** 2023-08-21

**Authors:** Tian Gan, Huanli An, Ming Tang, Hui Chen

**Affiliations:** State Key Laboratory for Conservation and Utilization of Subtropical Agro-Bioresources, College of Forestry and Landscape Architecture, South China Agricultural University, Guangzhou 510642, Chinazzm_ahl@stu.scau.edu.cn (H.A.); tangming@scau.edu.cn (M.T.)

**Keywords:** *Leptographium qinlingensis*, RNA interference, *Agrobacterium tumefaciens*, FlbA, pathogenicity

## Abstract

*Leptographium qinlingensis* is a pathogenic fungus of *Pinus armandii* that is epidemic in the Qinling Mountains. However, an effective gene interference strategy is needed to characterize the pathogenic genes in this fungus on a functional level. Using the RNA silencing vector pSilent-1 as a template, we established an RNA interference genetic transformation system mediated by *Agrobacterium tumefaciens* GV3101, which is suitable for the gene study for *Leptographium qinlingensis* by homologous recombination and strain interference system screening. The LqFlbA gene was silenced using the RNA interference approach described above, and the resulting transformants displayed various levels of silencing with a gene silencing effectiveness ranging from 41.8% to 91.4%. The LqFlbA-RNAi mutant displayed altered colony morphology, sluggish mycelium growth, and diminished pathogenicity toward the host *P. armandii* in comparison to the wild type. The results indicate that this method provides a useful reverse genetic system for studying the gene function of *L. qinlingensis*, and that *LqFlbA* plays a crucial role in the growth, development, and pathogenicity of *L. qinlingensis*.

## 1. Introduction

The *Pinus armandii*, commonly known as Chinese white pine, stands as a native and pioneering coniferous species within the Qinling Mountains of China [1]. It assumes a crucial ecological and societal responsibility in water and soil conservation, thus wielding a significant impact on the local economy and ecology [2]. However, a rapid, widespread decline in Chinese white pine (*P. armandii*) has been brought on by an interaction biological complex (CWPB-Lq complex) that is formed of the Chinese white pine beetle (*Dendroctonus armandi*, CWPB) and the ascomycetes fungus (*Leptographium qinlingensis*, Lq) [3,4,5]. The activities of *L. qinlingensis* culminate in disruptions of nutrient and water metabolism, eventually leading to the demise of host trees due to the destruction of resin-producing cells and the obstruction of resin canals [6]. Additionally, according to recent research, symbiotic fungi help beetles invade trees by detoxifying the chemical defenses of the trees and providing the insects with nourishment [7,8,9,10,11,12].

*L. qinlingensis* belongs to the ophiostomatoid fungi. Given the wealth of biological information available on this pathogenic CWPB-associate, it is the primary target in our current and ongoing genomics work. Li et al. (2012) reported three plant toxins and their activities in metabolites secreted by *L. qinlingensis* [1]. Dai et al. (2015, 2022) systematically studied the phylogenetic development of the CYP450 family genes in *L. qinlingensis* and their detoxification mechanism in overcoming pine defense compounds [13,14]. Furthermore, Dai et al. (2022) delved into the transcriptomic analysis of *L. qinlingensis*, revealing key genes and enzymes participating in terpene tolerance and nutritional utilization [8]. An et al. (2022) explored the intricate connection between the TOR gene and various factors such as carbon sources, nitrogen sources, host nutrients, and host volatiles (monoterpenoids) within *L. qinlingensis* [15]. Gan et al. (2022) reported on the role of RGS family genes in the fungal growth and development of, as well as in overcoming the host chemical resistance of, *L. qinlingensis* [4]. Nevertheless, the verification of gene functions and their contributions to pathogenicity necessitates the generation of mutants (or transformants) of *L. qinlingensis*, wherein the genes of interest are either deleted or modified. The methodologies for gene replacement and modification require tailored optimization for each fungal species, a task that has not yet been accomplished for *L. qinlingensis*.

RNA interference (RNAi) is a simple, quick, precise way of exploring gene function, applied to switching off genes in plants, animals, insects, and many other higher organisms [16]. Notably, RNAi has been successfully employed within numerous fungi [17,18,19,20,21,22,23]. Nakayashiki et al. (2005) pioneered the development of a restriction enzyme-based silencing vector, pSilent-1, tailored for RNA silencing investigations in filamentous fungi [18]. Meanwhile, the *Agrobacterium tumefaciens*-mediated transformation (ATMT) system is gaining popularity as an effective insertional mutagenesis method for gene overexpression, gene targeting, and T-DNA insertional mutagenesis in a variety of filamentous fungus [24,25,26]. In contrast to protoplast-mediated transformation (PMT), ATMT confers a discernible advantage, given its capacity to facilitate the direct exploitation of fungal spores for genetic manipulation, obviating the requisite for protoplasts [27]. Despite its widespread application in various filamentous fungi, the successful implementation of ATMT within the context of *L. qinlingensis* has yet to be realized. The pressing need revolves around establishing a dependable methodology for achieving stable genetic transformation in *L. qinlingensis*, thereby enabling the comprehensive characterization of the functional roles encoded by its virulence genes.

Within filamentous fungi, heterotrimeric G proteins regulate multiple physiological processes by regulating adenylate cyclase and phospholipase activities [28,29,30,31]. As a negative regulator of G protein signaling, the G protein signaling regulator (RGS) is involved in many physiological processes such as the growth and development of filamentous fungi. FlbA constitutes the inaugural scrutinized RGS protein within the domain of model filamentous fungi [32]. Extant research has incontrovertibly elucidated the pivotal role of the *FlbA* gene in governing the growth and development of numerous filamentous fungal species [33,34,35]. In the model fungus *Aspergillus nidulans*, *AnFlbA* regulates germination of conidia and tolerance of environmental stress [36]. The *MoRgs1* (*FlbA*) of *Magnaporthe oryzea* controls conidia and appressorium formation [31]. In *Fusarium verticillioides*, *FvFlbA2* has a negative regulation of conidial production [37]. In our previous study, the *FlbA* gene played a role in the growth and pathogenicity of *L. qinlingensis* [4]. In light of its gene functionality and discernible phenotypic alterations, the *FlbA* gene was selected as the designated target for knockdown in the context of this investigation.

In the work reported here, we established a genetic transformation system for *L. qinlingensis* by conjoining *A. tumefaciens*-mediated transformation (ATMT) with RNA interference (RNAi). Through the targeted manipulation of the *LqFlbA* gene, we achieved the generation of transformants characterized by gene silencing, thereby elucidating the influence of *LqFlbA* on fungal growth and pathogenicity. This account stands as the inaugural report detailing the achievement of a successful and stable genetic transformation within this species. Consequently, this accomplishment holds promising implications for the prospective advancement of genetic manipulation in closely related fungal taxa.

## 2. Results

### 2.1. Antibiotic Sensitivity of Experimental Strains

**Figure 1:**
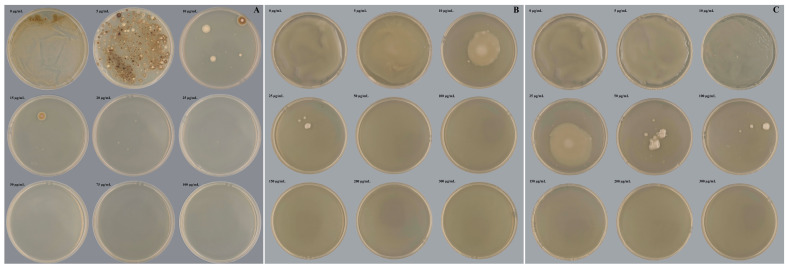
Antibiotic sensitivity of experimental strains. (**A**) Hygromycin B sensitivity of *L. qinlingensis*; (**B**) Cefotaxime sensitivity of *A. tumefaciens* GV3101; (**C**) Timentin sensitivity of *A. tumefaciens* GV3101.

Hygromycin B sensitivity in *L. qinlingensis* was evaluated by incubating the isolate on PDA supplemented with various concentrations of hygromycin B (up to 100 µg/mL) at temperatures of 28 °C for 7 days in darkness. Growth was fully suppressed at the hygromycin B concentration of 50 µg/mL (Figure 1A); consequently, this threshold was employed to select the resistant transformants in the context of ATMT experiments. Meanwhile, varying concentrations (up to 300 µg/mL) of cefotaxime or timentin exhibited no discernible impact on the growth of *L. qinlingensis*. Application of cefotaxime and timentin was undertaken to eliminate *A. tumefaciens* in the elective PDA medium. *A. tumefaciens* GV3101 growth was completely arrested at a cefotaxime concentration of 50 µg/mL (Figure 1B) and a timentin concentration of 150 µg/mL (Figure 1C); consequently, these specific concentrations were adopted for the eradication of *A. tumefaciens* in the ATMT experiments.

### 2.2. ATMT of L. qinlingensis

The effect of different cocultivation parameters (*A. tumefaciens* and *L. qinlingensis* concentrations, cocultivation duration, temperature, pH of the medium, and acetosyringone concentration) on the transformation efficiency of *L. qinlingensis* was evaluated with the pSilent-1 vector. The transformation rate was determined by calculating the number of transformants obtained under different parameter conditions.

**Figure 2:**
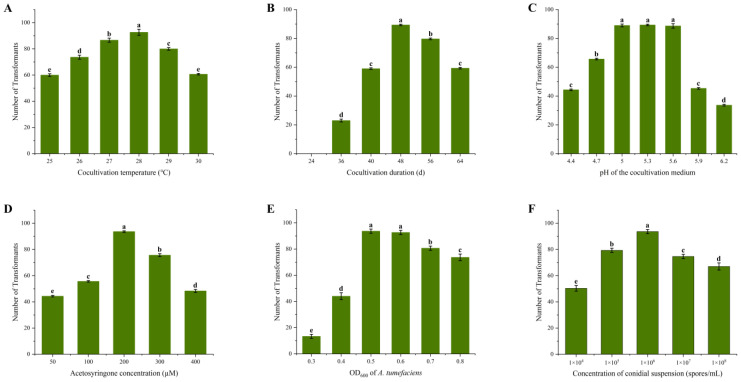
The effects of different cocultivation parameters on ATMT of *L. qinlingensis*. (**A**) Cocultivation temperature; (**B**) Cocultivation duration; (**C**) pH of the cocultivation medium; (**D**) Acetosyringone concentration; (**E**) The concentration of *A. tumefaciens* GV3101; (**F**) The conidia concentration of *L. qinlingensis*. The values are the means ± SE of three independent experiments. Error bars represent standard deviation from repeats. Different letters indicate a significant difference between different treatments (*p* < 0.05).

The study on the impact of six different cocultivation temperatures (viz.25, 26, 27, 28, 29, and 30 °C) on the transformation rate showed that higher numbers of transformants were achievable at 28 °C (Figure 2A). Among the tested cocultivation periods (24, 36, 40, 48, 56, and 64 h), a 48 h incubation period was optimal for obtaining a higher yield of transformants (Figure 2B). Lower transformation efficiency was noted with a longer cocultivation period. Among the 7 different pH values (pH 4.4, 4.7, 5.0, 5.3, 5.6, 5.9, and 6.2) of the cocultivation medium evaluated, higher numbers of transformants were noted at pH 5.0–5.6 (Figure 2C). Cocultivation performed with 200 µM acetosyringone yielded the highest transformation rate compared to the other tested concentrations (50, 100, 300, and 400 µM). A decrease in the number of transformants was noted with an increase in the acetosyringone concentration (Figure 2D). Among the tested cell concentrations of *Agrobacterium* (OD_600_ = 0.3, 0.4, 0.5, 0.6, 0.7, and 0.8) and conidial suspension(10^4^, 10^5^, 10^6^, 10^7^, and 10^8^ spores/mL), *Agrobacterium* (OD_600_ = 0.5–0.6) and conidial suspension (1 × 10^6^ spores/mL), respectively, were found to be ideal for generating higher numbers of transformants (Figure 2E,F).

Collectively, the transformation efficiency increased using the Agrobacterium (OD_600_ = 0.5–0.6) and conidial suspension (1 × 10^6^ spores/mL) in the cocultivation medium with a pH value of 5.0–5.6. The transformation should occur at 28 °C in the presence of 200 µM acetosyringone for no longer than 48 h to yield a high transformation efficiency.

### 2.3. Confirmation of Transformants

**Figure 3:**
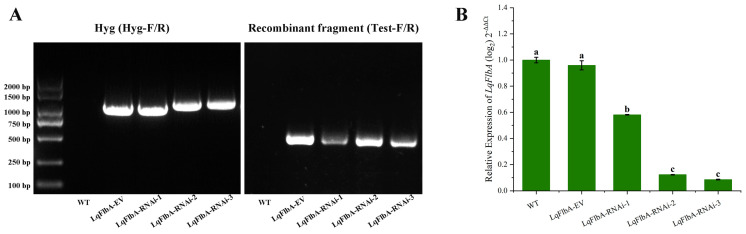
Molecular characterization of *FlbA* gene silencing transformants. (**A**) PCR amplification of recombinant fragment (484 bp), Hyg (1040 bp) using primers indicated in a from WT, LqFlbA-EV, and LqFlbA-RNAi transformants. (**B**) Transcriptional levels of LqFlbA in WT, LqFlbA-EV, and LqFlbA-RNAi transformants by real-time qRT-PCR analysis. Different letters indicate a significant difference between different treatments (*p* < 0.05).

To validate the accurate gene integration within the transformants, we selected three positive transformants (designated as LqFlbA-RNAi-1, LqFlbA-RNAi-2, and LqFlbA-RNAi-3), and juxtaposed them with a transformant harboring the vacant vector (referred to as LqFlbA-EV) following a series of five consecutive screening iterations. By employing specific primers (Hyg-F/R, Test-F/R) in PCR analyses, we successfully verified the presence of recombinant fragments and the hygromycin resistance (*Hyg*) gene. All three transformants, alongside LqFlbA-EV, exhibited the anticipated amplicons, namely a band corresponding to the recombinant fragment (484 bp) and a Hyg band (1040 bp), while displaying no bands for the wild type (WT). PCR products of the amplified *Hyg* gene were sequenced correctly (Figure 3A). Subsequent sequencing of PCR products using the Test-F/R primers revealed the presence of recombinant fragments in all three positive transformants, while no hairpin structures were detected in LqFlbA-EV.

To assess the efficacy of target gene silencing in the transformants, we employed specific primers (FlbA-F/R) and performed quantitative real-time PCR (qRT-PCR) to quantify the relative expression levels of the target genes across the three transformants and LqFlbA-EV. Compared with WT, the expression level of the *FlbA* gene was not significantly changed in LqFlbA-EV, while the *FlbA* gene expression level was significantly reduced in the three transformants, with gene silencing efficiency of 41.8%, 87.7%, and 91.4%, respectively (Figure 3B).

### 2.4. LqFlbA Is Required for the Growth and Reproduction of L. qinlingensis

**Figure 4:**
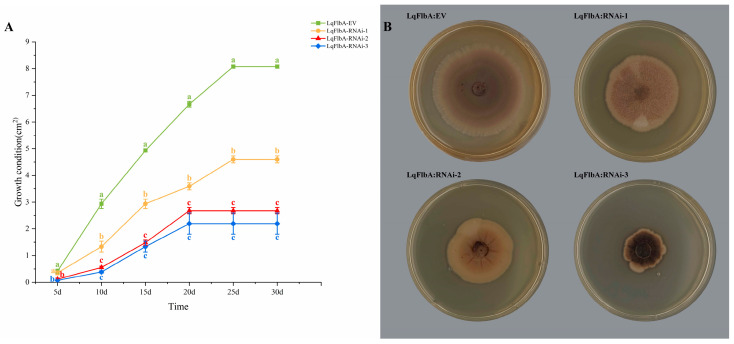
Effect of *LqFlbA* silencing on the growth and reproduction of *L. qinlingensis*. (**A**) Growth rate of LqFlbA-EV and 3 RNAi transformants. The growth condition was obtained by calculating the area of the colony. The results represent the mean ± SE of three independent experiments. Different letters indicate a significant difference between different strains (*p* < 0.05). (**B**) Phenotypes of LqFlbA-EV and 3 RNAi transformants on PDA plates (added 50 µg/mL hygromycin B).

To ensure consistent medium conditions, LqFlbA-EV was used as a control to compare hyphal growth after *LqFlbA* gene silencing. We detected that *FlbA* gene silencing caused the premature termination of colony growth in *L. qinlingensis* (Figure 4A). LqFlbA-RNAi-1, LqFlbA-RNAi-2, and LqFlbA-RNAi-3 stopped growing on the 25th, 20th, and 20th days, respectively, with colony areas of only 56.9%, 33.2%, and 27.2% of the control. Altogether, these findings reveal that the more efficient *FlbA* gene silencing, the lower the growth rate of the transformants and the more inhibited the colony growth.

Compared with the control (LqFlbA-EV), the colony morphology of the three RNAi transformants changed (Figure 4B). The mycelium of LqFlbA-RNAi-3 has more melanin deposition and a darker colony color. The mycelium growth of LqFlbA-RNAi-2 accumulates longitudinally, and the colonies exhibit longitudinal ridges that diverge from the center to the surrounding areas. LqFlbA-RNAi-1 showed white powdery hyphae free from the PDA plate, with uneven colony coloring. None of the three RNAi transformants colonies produced ring-like discoloration.

### 2.5. Knockdown of LqFlbA Results in Diminished Pathogenicity of L. qinlingensis

**Figure 5:**
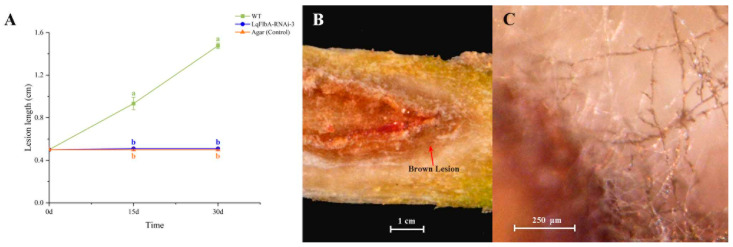
Effect of the *LqFlbA* on the pathogenicity of *L. qinlingensis*. (**A**) Disease spot length on *P. armandii* seedlings inoculated with WT and RNAi transformants. The wild type successfully colonized and grew on pine seedlings, while the LqFlbA-RNAi-3 and agar control did not produce a significant reaction. Each treatment was repeated three times with new sapling and fungal for each replicate. Different letters indicate a significant difference between different strains (*p* < 0.05). (**B**) The wild type induced a brown lesion in the phloem and sapwood. (**C**) Hyphe of *L. qinlingensis* growing near the diseased spot tissue.

To ascertain whether *FlbA* plays a pivotal role in the colonization and infection processes of pine trees, LqFlbA-RNAi-3 (exhibiting the highest silencing efficiency), the wild-type strain, and non-inoculated agar (serving as a control) were chosen for inoculation onto two-year-old *P. armandii* seedlings. Spot length was measured at 15 days and the fungi on the spot were reisolated and identified. The outcomes unequivocally revealed that the knockdown of *LqFlbA* led to a discernible reduction in the pathogenic potential of *L. qinlingensis* (Figure 5). At the culmination of a 15-day interval, the wild-type strain elicited a brown-hued lesion, measuring approximately 1 cm in length (averaged from three independent experiments), within the phloem and sapwood. In contrast, LqFlbA-RNAi-3 and the agar control treatments elicited a notably subdued response. Subsequent to a 30-day period, seedlings inoculated with the wild-type strains displayed a yellowing of their needles, accompanied by an approximately 1.5 cm long disease spot. Conversely, pine trees subjected to LqFlbA-RNAi-3 and the control exhibited robust health, with negligible discernible reactions at the inoculation site. Notably, both the wild-type and transformant variants were successfully isolated from the periphery of the inoculation site.

## 3. Discussion

Functional assessments of target genes in filamentous fungi conventionally necessitate the establishment of a robust and efficient genetic transformation system [38,39]. RNA interference (RNAi) is a sequence-specific post-transcriptional gene silencing phenomenon and has been used successfully for gene knockdown in filamentous fungi [18,40]. A central advantage inherent to RNAi resides in its non-invasive alteration of the genomic structure, unlike gene knockout methodologies, thus ensuring that the gene expression remains partially sustained rather than being entirely abrogated [41]. Nakayashiki et al. (2005) developed a restriction enzyme-based silencing vector, pSilent-1, for RNA silencing studies in filamentous fungi [18]. The pSilent-1 vector has been effectively used to investigate the role of genes in a variety of filamentous fungi. In *Metarhizium anisopliae*, pSilent-1 plasmid was used interfered with the *swnk* gene of the WT strain [42]. Using the pSilent-1 vector, the chr-1 gene was suppressed in *Neurospora crassa*, resulting in transformants that displayed resistance to chromate and reduced accumulation of chromium [43]. Scindiya et al. employed the RNA interference (RNAi) methodology via the pSilent-1 vector to systematically investigate the functional attributes of pathogenicity-associated genes (PKS1, GT, and SNF1), thereby elucidating their respective contributions to virulence within *Colletotrichum falcatum* [44,45]. The *A. tumefaciens*-mediated transformation (ATMT) method, characterized by enhanced stability and efficiency, surpasses conventional transformation methodologies in its applicability to fungal transformation processes [46,47]. The ATMT method has been applied to many ascomycetes [48,49,50,51,52,53]. To further validate the selected pathogenicity gene, attempts were made to standardize the RNAi-based approach during *L. qinlingensis* pathogenesis. Since our earlier study confirmed that *FlbA* plays a role in the growth and pathogenicity of *L. qinlingensis* [4], functional analysis of the *FlbA* gene has been selected for the RNAi approach using the fungal transformation vector pSilent-1 through *Agrobacterium*-mediated transformation (ATMT) and achieved knockdown of the target genes.

Many factors affect the ATMT efficiency, e.g., the type of starting fungal material (protoplast, spore, and hypha), bacterial and fungal cell concentrations, temperature, length of co-incubation, and concentration of acetosyringone, which is a plant metabolite released from wounded roots that enhances *A. tumefaciens* transformation [54,55]. The observed variations in transformation conditions can be attributed to fungal biodiversity. In this study, we investigated the factors that impact the efficiency of ATMT for *L. qinlingensis* (Figure 2 and Figure 3). Our findings revealed that *L. qinlingensis* is highly sensitive to hygromycin B. In comparison to other types of ascomycetes fungi, a lower concentration of hygromycin B (50 µg/mL) was sufficient to impede the growth of *L. qinlingensis* conidia (Table 1). This revelation holds the potential for strategic utilization in the chemical management of *L. qinlingensis*.

In filamentous fungi, RGS proteins play crucial roles in fundamental biological processes [28,32,65,66]. FlbA is only the second RGS protein to be identified, and plays a central role in attenuating heterotrimeric G-protein-mediated vegetative growth signaling in fungi [36]. In the model filamentous fungus *Aspergillus nidulans*, deletion of the *FlbA* results in the undifferentiated proliferation of hyphal mass and the absence of sexual/asexual sporulation as well as blockage of the mycotoxin sterigmatocystin (ST) production [67,68,69]. In order to corroborate the *FlbA* gene silencing among the transformants, the mutants underwent scrutiny via qRT-PCR assays for *FlbA* expression in the context of this study. The three transformants exhibited notable disparities, conceivably attributable to the pSilent-1 gene silencing vector. Due to the integrity of the transcriptional unit of target gene hairpin RNA varies in the transformant genome, the gene silencing efficiency varies in the transformants. The transformants were categorized as strongly silenced transformants (0–25%), moderately silenced transformants (50–75%), and non-silenced transformants (75–100%), based on the expression level of the target genes (compared to the wild type) [18]. In this study, LqFlbA-RNAi-2 and LqFlbA-RNAi-3 belonged to strongly silenced transformants, whereas LqFlbA-RNAi-1 belonged to moderately silenced transformants. Due to the intact hairpin structure in the strongly silenced transformants genome and the low risk of transgene loss, strongly silenced transformants were preferentially selected for the subsequent gene function validation. The results of this study showed that there were clear differences in the LqFlbA-RNAi transformants and WT in growth and pathogenicity. Knockdown of *FlbA* reduced the growth and pathogenicity of *L. qinlingensis*. These outcomes robustly corroborate the postulation that the RNAi and ATMT methodologies can effectively facilitate the isolation of functional genes and the identification of pathogenicity-associated genes within *L. qinlingensis*.

In conclusion, we developed an efficient genetic transformation system for *L. qinlingensis*, a bark beetle-vectored fungal pathogen associated with the Chinese white pine. The approach amalgamated *A. tumefaciens*-mediated transformation (ATMT) with RNA interference (RNAi) techniques. The procedure demonstrated notable efficacy, yielding transformants characterized by the *FlbA* gene knockdown within our wild-type *L. qinlingensis* strain. The elucidated genetic transformation system delineated within this study will serve as a pivotal tool for the functional elucidation of genes implicated in the pathogenicity of *L. qinlingensis*, thus facilitating a comprehensive understanding of the mechanisms through which the fungus can bypass or overcome tree defense mechanisms.

## 4. Materials and Methods

### 4.1. Strains, Plasmids, and Media

*L. qinlingensis* (NCBI Taxonomy ID: 717,526) was isolated from *P. armandii* sapwood phloem that had been attacked by the *D. armandi* in the Qinling Mountains and deposited at the College of Forestry and Landscape Architecture, South China Agricultural University (Guangzhou, China). *Escherichia coli* (DH5α) and *A. tumefaciens* (GV3101) were purchased from Shanghai Weidi Biotechnology Co., Ltd. (Shanghai, China). Plasmid pSlient-1 was purchased from Beijing Jiarui Hengtong Biotechnology Co., Ltd. (Beijing, China).

Potato dextrose agar (PDA) medium (2% glucose, 1.5% agar) was used for the cultivation of *L. qinlingensis*. When screening transformants, appropriate concentrations of antibiotics need to be added to the PDA culture medium. Luria–Bertani (LB) medium (0.5% yeast extract, 1% tryptone, 1% NaCl, solid LB medium with 1.5% agar) was used for the culture of *E. coli* and *A. tumefaciens* GV3101. Induction medium (IM) and co-cultivation medium (CM) were used for fungal transformation and required the addition of 0.2 mol/L acetosyringone (AS) [70].

### 4.2. Construction of Silencing Vectors

**Figure 6:**
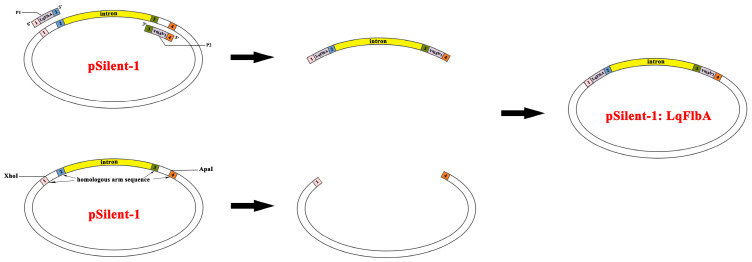
Schematic diagram for constructing silencing vectors. Double-enzyme digestion was performed at the XhoI and ApaI sites to obtain the pSlient-1 vector linearized fragment. A fragment containing one pair of reverse complementary silencing targets was obtained by PCR amplification using pSlient-1 vector as template and P1 and P2 as primers. The RNA silencing vector pSilent-1: LqFlbA was obtained by homologous recombination of the two fragments obtained as described above using the recombinant enzyme Exnase II.

The construction of the gene silencing vector was modified from the method of Zhong et al. [71]. XhoI and ApaI sites on pSlient-1 were selected as cleavage sites for double enzyme digestion, two linearized fragments of pSlient-1 were obtained, and the larger fragments were recovered and stored at −20 °C. The RNA silencing target of the *LqFlbA* gene was designed using DSIR (http://biodev.extra.cea.fr/DSIR/DSIR.html, accessed on 15 July 2023), and homologous arms of the XhoI and ApaI cleavage sites were added on both sides of the target. Using the pSlient-1 vector as a template, a fragment containing two reverse-complementary *LqFlbA* silencing targets (called a hairpin region) was amplified and stored at −20 °C. The fusion of the two aforementioned fragments was accomplished using the recombinant enzyme Exnase II (C112-02, Vazyme, Nanjing, China) for the execution of the homologous recombination, followed by the subsequent introduction of the resultant constructs into *E. coli* DH5α. Positive clones were meticulously chosen for PCR-based identification and subsequent sequencing, ensuring the validation of their accuracy. This rigorous validation procedure was undertaken to attain the conclusive construction of the RNA silencing vector pSilent-1: LqFlbA (Figure 6). The pSlient-1 vector without the hairpin region (called pSlient-1:EV) was used as the control (RNAi empty vector).

### 4.3. Antibiotic Sensitivity of Experimental Strains

The conidial suspension of *L. qinlingensis* (10^6^/mL, 100 µL) was grown on PDA medium with added different concentrations of hygromycin B (0, 5, 10, 15, 20, 25, 50, 75, and 100 µg/mL), cefotaxime (0, 25, 50, 100, 150, 200, 250, 300, and 350 µg/mL) and timentin (0, 25, 50, 75, 100, 150, 200, 250, and 300 µg/mL), and incubated at 28 °C, in darkness, for 7 d to monitor its sensitivity level. Antibiotics were purchased from Sangon Biotech (Shanghai) Co., Ltd. (Shanghai, China).

We inoculated *A. tumefaciens* GV3101 containing pSlient-1 (empty vector) into the LB liquid medium and oscillated for 200 r/min. Then, we uniformly coated 100 µL *A. tumefaciens* GV310 (OD_600_ = 0.5–0.6) onto the PDA medium with different concentrations of cefotaxime (0, 5, 10, 25, 50, 100, 150, 200, and 300 µg/mL) and timentin (0, 5, 10, 25, 50, 100, 150, 200, and 300 µg/mL) added, and incubated it at 28 °C, in darkness, for 7 d to monitor its sensitivity level.

### 4.4. A. tumefaciens-Mediated Transformation (ATMT)

We referred to the operating instructions for the method of transferring the RNA silencing vector (pSilent-1: LqFlbA or pSlient-1:EV) into the *A. tumefaciens* GV3101 receptive cells. The GV3101 strain (carrying the RNA silencing vector) was washed 3 times with 1 mL IM liquid medium, and then shaken at 200 r/min until OD_600_ = 0.5–0.6. After 7 days of cultivation on the PDA medium, the *L. qinlingensis* was rinsed with sterile distilled water to yield an asexual spore suspension. The spore suspension was passed through three layers of lens paper to exclude mycelial fragments. Following this, the spores underwent triple rinsing with IM liquid medium. Subsequently, the conidial suspension was adjusted to a concentration of 1 × 10^6^ spores/mL.

A 100 µL conidial suspension (1 × 10^6^ spores/mL) mixed with a 100 µL *A. tumefaciens* (OD_600_ = 0.5–0.6) was spread on the 0.45 mm pore nitrocellulose filter laid on the CM plates with the appropriate concentration of acetosyringone (200 µM), and incubated for 48 h at 28 °C. Subsequently, the nitrocellulose filters were transferred onto PDA selective plates containing suitable concentrations of hygromycin B (50 µg/mL), cefotaxime (50 µg/mL), and timentin (150 µg/mL). Upon the anticipated emergence of putative transformants, they were manually isolated and subsequently transferred onto fresh PDA selective plates containing the determined concentration of hygromycin B (50 µg/mL), as ascertained through the antibiotic sensitivity assay. These isolates were then incubated at a temperature of 28 °C. After 5 consecutive screenings, positive transformants were obtained to ensure genetic stability. The GV3101 strain carrying the pSlient-1 served as the control (empty vector) in the ATMT experiment.

### 4.5. Confirmation of Transformants

Genomic DNA was extracted from *L. qinlingensis* using the Fungal DNA kit (D3390-01; Omega, Norcross, GA, USA) according to the manufacturer’ s protocol. Using genomic DNA as a template, the primers Hyg-F and Hyg-R were designed for PCR amplification of 1040 bp *Hyg* genes, and the primers Test-F and Test-R were designed on both sides of XhoI and ApaI sites for PCR amplification of 484 bp recombinant fragment (hairpin region) on pSilent-1: LqFlbA.

Total RNA was extracted from *L. qinlingensis* using a UNIQ-10 Column Trizol Total RNA Isolation Kit (B511321; Sangon Biotech, Shanghai, China) according to the protocol provided by the manufacturer. The first-strand cDNA synthesis was initiated using a HiScript^®^ III Q RT SuperMix for qPCR (+gDNA wiper; Vazyme, Nanjing, China) transcription kit. To quantify the expression level of the *LqFlbA* gene in the transformants, the primers for FlbA-F and FlbA-R were designed for qRT-PCR using cDNA as the template and the *LqEF1* gene (accession number: AHZ56579.1) as the reference gene. All the reactions were performed with three technical replicates of three biological replicates. The relative expression levels of the genes were computed by the 2^−ΔΔCt^ method of relative quantification [72]. The gene-specific primers of the research are shown in Table 2.

### 4.6. Optimization of Transformation Parameters

The evaluated cocultivation parameters that are known to influence transformation frequency included *Agrobacterium* cell concentration (OD_600_ = 0.3, 0.4, 0.5, 0.6, 0.7, and 0.8), conidial suspension concentration (10^4^, 10^5^, 10^6^, 10^7^, and 10^8^ spores/mL), cocultivation time (24, 36, 40, 48, 56, and 64 h), cocultivation temperature (125, 26, 27, 28, 29, and 30 °C), pH of the cocultivation medium (4.4, 4.7, 5.0, 5.3, 5.6, 5.9, and 6.2), and acetosyringone concentration (50, 100, 200, 300, and 400 µM). Based on the preliminary experiments’ findings, one parameter was varied while others were kept constant to evaluate each cocultivation condition’s effect on transformation. Following cocultivation, the cells were treated with cefotaxime (50 µg/mL) and timentin (150 µg/mL) for 48 h to eliminate *Agrobacterium*. The transformation rate was determined by calculating the number of transformants obtained under different parameter conditions. Each cocultivation parameter was examined in triplicate in at least three independent experiments.

### 4.7. Growth and Pathogenicity Assay of Transformants

We verified the effect of *FlbA* on the growth of *L. qinlingensis* by measuring the growth rate and colony morphology of the transformants. The three transformants were incubated on the PDA plate containing hygromycin B at 28 °C for 7 days. We drilled holes in the edges of the PDA plate and inoculated fresh mycelia plugs into the center of the new PDA plate. We measured the colony radius and recorded the colony morphology every 5 days under dark cultivation at 28 °C. The transformant carrying the empty vector (LqFlbA-EV) served as the control in this experiment. Each treatment was repeated three times.

Fungal inoculation into *P. armandii* stems was modified from the methods in Pham et al. (2014) [73]. Wild-type (WT) and transformant variants with the highest silencing efficiency (LqFlbA-RNAi-3) were selected for pine sapling inoculation experiments. We used non-inoculated agar as a control for the inoculation effect. Ten saplings were inoculated with each strain. Holes were drilled with a 0.5 cm cork borer at 4 cm above the soil line. Mycelia plugs of the wild type, transformants, and non-inoculated agar (control) were placed into the holes, and the bark was replaced over the mycelial plugs to close the inoculation holes. All the holes that were drilled for fungal inoculation were wrapped with cling wrap and sealed with duct tape to avoid contamination. Each treatment was repeated three times with new saplings and fungus for each replicate. The saplings’ phloem diseased spot length was measured every 15 days.

### 4.8. Statistical Analysis

All the data were presented as averages ± SE. The statistical analysis package SPSS 22.0 (SPSS Inc., Chicago, IL, USA) was used to perform statistical analyses. The one-way ANOVA with Tukey’s test was used for multiple comparison analyses. A value of *p* < 0.05 was considered to be statistically significant. The different letters on the graphs indicate significant differences among the treatments. Line charts and histograms were drawn with OriginPro 2021 software (OriginLab, Northampton, MA, USA). PCR products were scanned under the imaging system (ChemiDoc XRS+; Bio-Rad, Hercules, CA, USA). Photos of colonies and lesions were taken by stereomicroscope (DS-R12; Nikon, Tokyo, Japan). The schematic diagram of the vector construction and group diagram was drawn using Adobe Photoshop CS6 (Adobe, San Jose, CA, USA).

## Figures and Tables

**Table 1:** Sensitivity to hygromycin B of pathogenic ascomycetes fungi.

Ascomycetes Fungi	Host Tree	Sensitivity to Hygromycin B
*L. qinlingensis*	*P. armandii*	50 μg/mL
*Neonectria galligena*	Broad-leaved tree species	50 μg/mL [56]
*Ophiostoma piceae*	Coniferous trees	250 μg/mL [57]
*Ophiostoma ulmi*	Elm	200 μg/mL [58]
*Valsa mali var. mali (Vmm)*	Apple tree	60 μg/mL [59]
*Sphaerulina musiva*	*Populus trichocarpa*	50 μg/mL [60]
*Corynespora cassiicola*	Hevea brasiliensis	100 μg/mL [61]
*Monilinia fructicola*	Stone and pome fruit	100 μg/mL [62]
*Dothistroma septosporum*	Coniferous trees	100–200 μg/mL [63]
*Raffaelea lauricola*	*Persea palustris* and *Persea americana*	110 μg/mL [64]

**Table 2:** Primer sequences used in the research.

Primer Name	Sequences (5′→3′)	Purpose
P1	catcgataccgtcgaccGCAGCGAGGATATTTCCAAGAagcttgctggaggatac	Construction of silencing vectors
P2	acgttaagtggatccggGCAGCGAGGATATTTCCAAGAgtaccacaggccttagca	
Hyg-F	ATGAAAAAGCCTGAACTCAC	Confirm transformants
Hyg-R	GGTCGGCATCTACTCTATTC	
Test-F	AGGAACGAGGACATTATT	Confirm transformants
Test-R	GCTGACATCGACACCAAC	
FlbA-F	GCTATGATCGACACCCTGAAG	qPCR
FlbA-R	GTAGTTTCTCAGCGTATGGTCG	
EF1-F	CCGCTGGTACGGGTGAGTT	qPCR
EF1-R	CTTGGTGGTGTCCATCTTGTT	

## Data Availability

Data is contained within this article.
